# Rebuilding the foundation: recommendations from the Summit to Revitalize Primary Care (Rev PC)

**DOI:** 10.1186/s12919-026-00364-6

**Published:** 2026-03-02

**Authors:** The Summit to Revitalize Primary Care Writing Committee*, Anthony Jerant, Richard L. Kravitz, Joshua J. Fenton, Courtney R. Lyles, Kevin Grumbach, Mark C. Henderson, Dominique Ritley, Margareta Brandt, Deborah Cohen, Erik Fernandez Y. Garcia, Kathryn E. Phillips, Russell S. Phillips, Diane Rittenhouse, S. Monica Soni, Lemeneh Tefera, Raymond Tsai, Ilana Yurkiewicz

**Affiliations:** 1 Department of Family and Community Medicine, University of California Davis School of Medicine, Sacramento, CA USA; 2 Division of General Medicine, Department of Internal Medicine, University of California Davis School of Medicine, Sacramento, CA USA; 3 University of California Davis Center for Healthcare Policy and Research, Sacramento, CA USA; 4 Department of Public Health, University of California Davis School of Medicine, Sacramento, CA USA; 5 Department of Family and Community Medicine, University of California San Francisco School of Medicine, San Francisco, CA USA; 6 Office of Health Care Affordability, California Department of Health Care Access and Information, Sacramento, CA USA; 7 Department of Family Medicine, Oregon Health & Science University School of Medicine, Portland, OR USA; 8 Department of Pediatrics, University of California Davis School of Medicine, Sacramento, CA USA; 9 California Health Care Foundation, Oakland, CA USA; 10 Division of General Medicine, Department of Medicine, Harvard Medical School, Boston, MA USA; 11 Mathematica, Oakland, CA USA; 12 Covered California, Sacramento, CA USA; 13 Division of General Medicine and Health Services Research, Department of Medicine, University of California Los Angeles School of Medicine, Los Angeles, CA USA; 14 California Department of Health Care Access and Information, Sacramento, CA USA; 15 Purchaser Business Group On Health, Concord, CA USA; 16 Division of Primary Care and Population Health, Department of Medicine, Stanford University, Palo Alto, CA USA

**Keywords:** Continuity of patient care, Patient-centered care, Primary health care, Health equity, Health care financing, Physician workforce planning, Health care costs, Health services research

## Abstract

In 2021, an ad hoc committee of the United States (U.S.) National Academies of Science, Engineering, and Medicine (NASEM) affirmed that robust, relationship-centered primary care is the foundation of efficient, effective health care. Yet the ad hoc committee also noted primary care was “slowly dying,” due to chronic under-investment, ill-suited payment models, and inadequate workforce planning and development. Encouragingly, efforts to revitalize primary care are underway. To accelerate this movement by generating expert consensus recommendations on the highest priority actions to take in repairing the frayed U.S. primary care base, clinical scientists at the University of California Davis (UCD) School of Medicine convened the Summit to Revitalize Primary Care (Rev PC). Summit recommendations were generated in four closed working sessions of a national Expert Committee. Committee members were selected to ensure a breadth of perspectives (e.g., health plans, purchasers of insurance, regulatory agencies, health systems, clinicians, educators, researchers, economists) from the public and private sectors. Seven high priority recommendations emerged: (1) Increase the proportion of spending on primary care, coupled with initiatives to slow the growth in total health care spending; (2) Pay for primary care using models that support high quality, team-based, relationship-centered, equitable care; (3) Assist practices in transformation to advanced primary care models and assess the impacts on clinical teams, patients, and communities; (4) Maximize primary care’s potential to equitably advance health; (5) Advocate for training an appropriately large and diverse primary care physician workforce; (6) Expand research to address the most pressing issues in primary care; (7) Collaborate with a broad array of societal stakeholders in messaging the importance of robust primary care. These recommendations both overlap with and expand on those of the NASEM ad hoc committee and subsequent Standing Committee on Primary Care. Broad pursuit of the recommendations would catalyze sustained momentum toward appropriate primary care investment and workforce planning and development, enabling U.S. primary care to realize its yet-unfulfilled potential to improve population health and advance health equity while helping to control growth in total health care costs.

## Introduction

A landmark 2021 United States (U.S.) National Academies of Science, Engineering, and Medicine (NASEM) report affirmed that robust, relationship-centered primary care is the foundation of an optimally efficient and effective health care system [1]. In the report, high quality primary care was defined as:

“….the provision of whole person, integrated, accessible, and equitable health care by interprofessional teams who are accountable for addressing the majority of an individual’s health and wellness needs, across settings and through sustained relationships with patients, families, and communities” [1].

“Whole person” care is concerned with well-being rather than merely the absence of disease, and considers mental, physical, emotional, and spiritual health as well as social influences on health. Research from several countries has indicated greater exposure to primary care is associated with better population health and longevity [2, 3]. For this reason, the NASEM report further designated primary care as a *common good* benefitting society and individuals in ways that other elements of the health care system do not [1, 4].

However, in recent decades it has become increasingly clear that U.S. primary care is in crisis, to the detriment of individual health, population health, and cost-effectiveness of health care [1–9]. Indeed, the NASEM report indicated that primary care in the U.S. is “slowly dying,” due to a nexus of factors including chronic and severe under-resourcing by health insurers and health systems, inadequate payment models to support it, and inattention to health care workforce planning and policy efforts [1]. As such, the NASEM high-quality primary care definition remains largely aspirational, with most practices lacking one or more of the core primary are attributes.

While the crisis is undeniable, the highest priority steps to take to ensure optimal primary care will be available to all in the U.S. have been less clear. Generating expert consensus recommendations on the highest priority actions was the focus of the Summit to Revitalize Primary Care (Rev PC), convened by the University of California Davis (UCD) School of Medicine in October 2024. In this report, we first briefly review the context for undertaking Rev PC and outline its activities. We then summarize the recommendations for highest priority actions to revitalize primary care generated by a committee of national experts convened at the Summit. While the focus of the Summit was on the U.S., we anticipate many of the recommendations may have relevance elsewhere [10, 11].

## Key drivers and manifestations of the U.S. primary care crisis

The primary care spending rate is the amount of money spent on primary care services as a proportion of overall (total) health care spending [12]. On average, the primary care spending rate in the U.S. is less than 5 cents out of every dollar spent on health care and declining, even though 35% of all health care visits and more than 50% of outpatient visits annually are to primary care clinicians, and primary care influences most other health care costs through related referrals, testing, prescription medications, procedures, and hospitalizations [7, 9]. The prevailing fee-for-service model of health care payment in the U.S. compounds the problem, supporting care for discrete patient visits with physicians and other advanced practice professionals (e.g., nurse practitioners, physician assistants, psychologists) rather than the whole-person focused, team-based models needed to provide top-quality primary care, which incorporate community health workers, social workers, dieticians, and pharmacists among others [13]. A further contributor to the under-resourcing of primary care is the deeply flawed methodology the U.S. Centers for Medicare and Medicaid Services (CMS) uses to determine Relative Value Units (RVUs) and fee schedules for different types of clinical work. The methodology severely undervalues the cognitive tasks that represent the lion’s share of primary care work (e.g., diagnosis and behavioral and medication management of health conditions) while overvaluing procedural (e.g., surgical) interventions [14, 15]. The result is that primary care practices are not adequately supported to deliver the optimal care needed to achieve the best patient outcomes [16]. Partly for these reasons, population health is poorer in the U.S. than in other comparably developed nations, which spend less on health care but considerably more on primary care as a proportion of total health care spending [6].

The low valuation of cognitive primary care services by the American Medical Association Specialty Society Relative Value Scale Update Committee (RUC) – advisory to CMS—has also contributed to the salaries of family physicians, general internists, and pediatricians being among the lowest for all specialties in the U.S. [17]. This creates a strong disincentive for trainees to pursue primary care [17, 18]. Low payment relative to other specialties and spiraling clinical and administrative burdens resulting from mounting preventive care and chronic disease management recommendations, poor electronic health record interfaces, prior authorization requirements, and other factors have led to all-time low job satisfaction ratings and high rates of burnout among primary care clinicians [8, 19, 20]. This has prompted increasing numbers of primary care clinicians to limit their spectrum of practice, work only part time, leave clinical medicine for other roles, or retire early [21, 22]. Apart from directly contributing to a shortage of primary care clinicians, such choices also have indirect adverse impact, as medical trainees hear about and observe the disenchantment of primary care clinicians and elect to pursue other specialties [9, 23–25]. Together, these issues further exacerbate a longstanding shortage of primary care clinicians, which has gone largely unaddressed because the two entities that fund most graduate medical education in the U.S. – the Centers for Medicare and Medicaid Services and the Veterans Administration – have little accountability to ensure the production of an appropriately sized primary care workforce [26, 27].

The net impact of these issues has been a sharp rise in the number of Americans who lack a usual source of health care, with individuals in lower socioeconomic urban and rural areas most affected, further worsening population health and health disparities [9]. Among patients who do have a primary care clinician, many are dissatisfied with the timeliness and experience of care, in part because chronic underinvestment in primary care means many practices lack adequate resources and staffing [28–30].

## Prospects for primary care recovery

Although the outlook for primary care may seem dire, hope remains. Legislative and regulatory efforts are underway in a growing number of U.S. states and at the federal level to allocate a greater share of health care spending to primary care and implement more appropriate payment models to support robust, relationship-centered primary care consistent with the aspirational NASEM definition [5, 31–37]. Many of these efforts have been coupled with initiatives to slow the rate of growth in total health care spending, which is unsustainably high across the U.S. and has the best chance of being controlled through greater emphasis on high value primary care services. Concurrently, State and federal policy initiatives aim to increase the number of medical trainees pursuing primary care, particularly in communities with least access [25, 38].

## The Summit to Revitalize Primary Care (Rev PC)

While burgeoning state and federal efforts to revitalize primary care have created a more hopeful outlook, the highest priority steps to repair this foundational element of the U.S. health care system have remained unclear. Generating national expert consensus recommendations for these highest priority actions was the primary aim of the Summit to Revitalize Primary Care (Rev PC), held at the UCD School of Medicine, October 16–18, 2024.

Here, we provide a high-level summary of the Summit activities and conduct. Full details of the Summit activities and process for generating Expert Committee consensus high priority recommendations can be found at: https://health.ucdavis.edu/family-medicine/news-events/optimizing-the-primary-care-spend-symposium/ [39].

The Summit was composed of six open-to-the-public sessions and five closed Expert Committee working sessions. Of the public sessions, five were plenary presentations, while the sixth provided brief comments by a local California State Assemblymember active in health care legislation. The public sessions were included to help ensure a shared basic understanding among attendees of: (1) Key contributors to the primary care crisis; (2) Potential approaches for addressing the crisis; and (3) Important yet poorly understood issues and unanswered questions that, unless attended to, may hinder progress in revitalizing primary care.

The five closed Expert Committee working sessions were programmed and designed to generate consensus highest priority recommendations for the revitalization of U.S. primary care. These sessions convened 29 nationally recognized primary care thought leaders. The Expert Committee members were selected to represent a range of perspectives, including health plans, purchasers of health insurance (e.g., employers), health care delivery systems, clinicians, researchers, educators, advocates, and economists, with members drawn from both the public and private sectors.

The first Expert Committee session was primarily intended to serve as an introductory “icebreaker”, with brief (5 min or less) remarks by five pre-designated Committee members followed by informal group discussion. The remaining four Expert Committee sessions were structured working sessions in which the members deliberated on key issues contributing to the primary care crisis and then generated consensus highest priority recommendations for addressing them. Two of the four working sessions employed the Nominal Group Technique to generate consensus high priority recommendations [40]. The other two working sessions employed a less formal facilitation approach, though the facilitators took care to ensure that all members had input. Table 1 summarizes the focus topics and approaches for each Expert Committee session.

**Table 1:** Rev PC Expert Committee Sessions

Session Title/Topic	Conduct
Session 1: Sharing What Has Been Learned Through Efforts to Revitalize Primary Care	Five discussants – pre-selected by the organizers from the larger Committee to represent a range of backgrounds and perspectives – had 5 min each to share key experiences and observations about primary care revitalization. This was followed by a moderated discussion segment that afforded all Committee members the opportunity to react to and expand on the discussant comments
Session 2: How Should Practices be Paid to Provide Optimally Resourced, High-Quality, Relationship-Oriented Primary Care?	The Committee was divided into five smaller groups, each then considering two questions from the perspective of a hypothetical committee of payors, policymakers, and primary care clinicians charged with designing optimal hybrid payment models for primary care: • Question 1: How can hybrid payment models best be designed to help practices deliver high-quality primary care consistent with the 2021 NASEM report aspirational definition? • Question 2: How can fairness and equity across practices be ensured under hybrid payment for primary care, given substantial baseline differences in practice characteristics and patient populations? The full Committee then reconvened for brief small group report-outs on the highest priority recommendations, which were subsequently summarized by the session leads
Session 3a: Advancing Optimally Resourced, Relationship-Oriented Primary Care—Identifying Research that Must Be Conducted to Attain the Vision	This session ran concurrently with Session 3b (half of the Expert Committee members were pre-selected to participate in each session). The participants considered three interrelated discussion topics: • Discussion topic 1: The strengths in the existing primary care research base • Discussion topic 2: Gaps in the research base and challenges for future primary care research • Discussion topic 3: Re-envisioning primary care research for the future Participants first paired off to discuss their thoughts on each question, then individually recorded their thoughts on different colored sticky notes that were placed on a board at the front of the room. Then, all discussed the results as a group. Highest priority recommendations and thematic categories were summarized by the session lead
Session 3b: Advancing Optimally Resourced, Relationship-Oriented Primary Care: Inputs to Impact—Traversing the Gap Between Primary Care Funding and Transformation	This session ran concurrently with Session 3a (half of the Expert Committee members were pre-selected to participate in each session). The participants were further subdivided into 2 groups, each considering a specific question • Subgroup 1 question: What should we be measuring and monitoring to determine whether new resources aimed at primary care transformation are making their way from payers to health plans to primary care practices, ultimately benefiting patients and communities and supporting the primary care workforce? • Subgroup 2 question: What political and persuasive strategies on the local, state, and national levels should be adopted to maximize the probability that increased primary care spending targets are not derailed and that the new resources available to primary care will result in a more accessible, satisfying, and defragmented experience for patients and health care professionals? Both subgroups were conducted using the Nominal Group Technique [40], yielding priority-ranked lists of recommended approaches and practices
Session 4: Leveraging the California Office of Healthcare Affordability’s Increased Primary Care Spending Target to Advance Health Equity	In this session the Expert Committee was divided into four smaller groups, each of which considered the following question: • What will need to happen for the new California Office of Health Care Affordability (OHCA) increased primary care spending benchmark to lead to the improvements in health care and health that essentially all in the state need, while also reducing or eliminating the state’s most pressing health and health care disparities? The facilitators clarified that the question related to actions that may represent either *best practices* (demonstrated by experience or research to effective), or untested but promising approaches, representing research gaps to meriting further study. Three of the four subgroups were conducted using the Nominal Group Technique [40], yielding priority ranked lists of recommended approaches and practices

Following the Rev PC Summit, the Summit conveners (AFJ and RLK) reviewed the highest priority consensus recommendations from each of the Expert Committee working sessions and compiled a draft of the Summit recommendations. The draft was then sent to all the Expert Committee members for review and editorial suggestions. After reviewing and incorporating suggestions, the conveners created the final recommendations.

## Expert committee consensus high priority recommendations for primary care revitalization

From the combined deliberations in the Summit’s five closed Expert Committee working sessions, seven consensus high priority recommendations emerged to inform efforts to revitalize primary care, each with related sub-recommendations (Table 2). While some of the recommendations and sub-recommendations name specific accountable target entities, the Expert Committee members were not instructed or required to target their recommendations to specific entities, so most do not specify targets.

**Table 2:** Recommendations and sub-recommendations of the Rev PC Expert Committee

**1. Rebalance health care spending to increase the proportion earmarked for primary care services and reduce spending on other services**	
1.A. Encourage the development and consistent use of a uniform definition of primary care in which continuity, comprehensiveness, and coordination of care are emphasized and incorporated in tracking spending on primary care 1. B. Set and enforce higher primary care spending rate targets: at least double the current average 1.B.(1). Employ a gradual implementation approach to attaining primary care and total healthcare spending targets 1. C. Establish and enforce primary care spending accountability mechanisms, including by: 1.C.(1). Developing and implementing a public dashboard of health plan and health system primary care spending, to foster transparency and encourage adherence 1.C.(2). Empowering oversight and regulatory bodies to levy fines and develop health plan and health system performance improvement plans if primary care spending targets are not met 1.C.(3). Requiring health systems to report on the flow of money earmarked for primary care (e.g., clinical revenue, capitation dollars, quality incentive payments) through the system	
**2. Pay primary care clinicians and practices using models that support teams in delivering high quality, equitable, relationship-centered care**	
2.A. Develop and implement hybrid payment models in which a majority of payment is provided as per member per month capitation and a lesser proportion as fee-for-service payment 2.A.(1). Ensure hybrid payment models are risk-adjusted to guide the determination of appropriately sized patient panel sizes for clinicians, and to ensure there is sufficient support for care teams designed to meet the needs of the populations served 2.A.(2). Consider unique hybrid payment models for three categories of practices: Small, independent practices, community clinics (e.g., Federally Qualified Health Centers), and larger practices within integrated networks 2.B. Ensure a reliable and meaningful means of attributing patients to primary care practices 2.C. Consider establishing state primary care stabilization funds, funded by all payers and sequestered from the larger health system budget, to fund all primary care services 2.D. Develop a more objective and valid methodology for the valuation of medical services than the current system based on Relative Value Units (RVUs)	
**3. Assist practices in transformation to advanced primary care models and assess the impacts on clinicians, practices, patients, and communities**	
3.A. Incentivize and hold practices and health systems accountable for delivering high value primary care while minimizing the reporting burden by: 3.A.(1). Prioritizing monitoring of over-arching metrics that reflect core attributes of primary care including patient access to care via multiple modalities (e.g., in-person visits, asynchronous and synchronous telehealth, artificial intelligence [AI] platforms) and continuity, comprehensiveness, coordination, and patient centeredness of care 3.A.(2). Selecting and monitoring a manageably small number of condition-focused quality metrics that also reflect core primary care attributes 3.B. Monitor the impact of advanced primary care practice at the broader community level 3.C. Provide supplemental funding and technical consultation and assistance to help practices effectively and rapidly implement advanced primary care models including: 3.C.(1). Training and leveraging extended primary care team members such as community health workers to increase the reach of practices and support health and wellness in the communities they serve, 3.C.(2). Adopting AI and other technologies to reduce clinician administrative burden around charting, coding, and clinical decision-making, and 3.C.(3). Integrating behavioral health services, of paramount importance given the level of unmet in the U.S. and the impracticality of meeting the need via other channels	
**4. Maximize the impact of primary care as a lever for equitably advancing population health**	
4.A. Establish universal population health goals and employ multiple strategies in communicating and pursuing them, targeted to specific health system and societal structural barriers that adversely affect health and health care but to different degrees and ways across diverse groups of patients 4.B. Develop, implement, and enforce mandates for all ambulatory practices (primary care and subspecialty care) to participate in the care of patients with Medicaid insurance 4.B.(1). Explore the provision of additional payments (e.g., determined by risk adjustment) to practices that disproportionately care for the medically underserved 4.C. Remove cost sharing (copays and deductibles) for primary care services 4.D. Ensure that risk adjustment methodologies account for both clinical factors and social influences on health 4.E. Ensure adequate information technology and infrastructure (e.g., data exchange capabilities between relevant entities) to support ongoing measurement, monitoring, and public reporting of health equity-related data	
**5. Advocate for the training of an appropriately sized and diverse primary care physician workforce**	
5.A. Advocate for: 5.A.(1). Centers for Medicare and Medicaid Services to develop and publicly report on adherence to policies that ensure distribution of graduate medical education (GME) funding is proportionate to regional community needs 5.A.(2). State governmental use of Medicaid funds to support GME in ways that better meet the needs of all communities, such as training in ambulatory facilities (e.g., Federally Qualified Health Centers) in rural and urban medically underserved areas, and 5.A.(3). Community governance structures that hold Medicare and other GME funders (e.g., states) accountable for ensuring that primary care workforce needs are met in all regions	
**6. Expand resources and infrastructure to facilitate research addressing questions of pressing relevance to primary care and its revitalization**	
6.A. Increase federal and other funding to support primary care research, with a focus on new funding streams to support rigorous examination of topics that reflect the full complexity and cross-cutting nature of primary care 6.B. Broaden the reach, scope, applicability, and impact of primary care research by developing: 6.B.(1). Hub and spoke geographic networks of practices and investigators co-designing research studies 6.B.(2). A national longitudinal registry of primary care practices and core practice data elements to support larger scale collaborations and research 6.C. Develop and disseminate robust gold standard measures reflective of primary care attributes and elements – such as patient-centeredness, primary care team-centeredness, and primary care practice characteristics (e.g. patient panel sizes), to increase validity within studies and comparability across studies 6.D. Develop and consistently apply more advanced analytic approaches (e.g., parallel mixed methods, rigorous risk adjustment) better suited to the complexity of primary care and related research questions 6.E. Build more effective strategies to disseminate primary care research evidence into clinical practice	
**7. Engage, educate, and collaborate with a broad array of societal stakeholders in messaging the vital importance of robust primary care to population health and health equity**	
7.A. Engage, educate, and facilitate the building of coalitions of stakeholders in a strong U.S. primary care base 7.B. Develop a robust communication strategy which clearly conveys that all in the U.S. are poorly served by primary care in its current state, though some groups and communities are more poorly served than others 7.C. Create and disseminate a library of evidence-based primary care marketing resources via a hub and spoke model, to provide more consistent and powerful messaging about the importance of primary care to the U.S. population 7.D. Consider efforts to increase health and primary care literacy at the population level (e.g., via inclusion of elements in standard elementary through high school curricula) 7.E. Strongly encourage community governance structures for all primary care practices	

### Recommendation 1

Rebalance health care spending to increase the proportion earmarked for primary care services and reduce spending on other services. There was broad agreement among Expert Committee members that while increasing the proportion of spending by health plans and health systems on primary care services alone will not be *sufficient* to revitalize primary care, it is a necessary step of fundamental importance. Further, the Committee recognized that such efforts must be tied to broader efforts to slow down the growth in overall health care spending and improve consumer affordability. The rate of growth in health care spending in the U.S. is widely viewed as unsustainable and a major threat to the national economy. Rebalancing care spending by earmarking more funding for primary care services and proportionately less to other types of care could be effective in addressing this pressing problem [41].

#### Sub-recommendations

A. Encourage the development and consistent use of a uniform definition of primary care in which *continuity, comprehensiveness, and coordination* of care are emphasized and incorporated in tracking spending on primary care. The Expert Committee recognized that uniformity in definition will be important in ensuring fairness and comparability among regions and health systems. They further underscored that while continuity, comprehensiveness, and coordination of care are essential elements to reaping the full benefits of primary care [42–46], currently these attributes are downplayed or ignored by most U.S. health system and health plans in their conceptualizations of primary care. Instead, most focus largely on maximizing access to a source of primary care [47, 48]. Including comprehensiveness, continuity, and coordination of care in a uniform definition of primary care and linking health plan payments to related tracking metrics (e.g., indices of continuity with the assigned primary care clinician) would incentivize health systems and practices to build and maintain strength in all of the core attributes. The Expert Committee also emphasized that once a uniform definition of primary care is implemented, subsequent ad hoc adjustment of the definition (e.g., removal of certain attributes) by health plans and health systems must be strictly prohibited. Such an approach would prevent health plans and health systems from meeting primary care spending targets by focusing largely or solely on expanding access.
B. Set and enforce higher primary care spending rate targets: at least double the current average The Expert Committee recognized that increasing primary care spend rates to this level would bring the U.S. more in line comparable industrialized countries, which have better population health outcomes and lower total health care spending [6].
  1. Employ a gradual implementation approach to attaining primary care and total healthcare spending targets. This recommendation is intended to help ensure that most or all health plans and systems in a given region will have enough time to attain the targets, which would be expected to take several years. The recently implemented California Office of Healthcare Affordability (OHCA) Primary Care Investment Benchmark has incorporated this approach. It established both: (a) an annual improvement benchmark of 0.5 −1.0 percentage point per year increase in primary care spending as a percentage of total medical expense for each payer from 2025 through 2033; and (b) a statewide investment benchmark of 15% of total medical expense allocated to primary care for all payers by performance year 2034 [49]. OHCA views the increase in primary care investment as a pivotal element of its concurrent effort to slow the rate of growth in total health care spending in California, which in recent years has surpassed the already high national rate of growth [49]. Several other states are pursuing similar phased multi-year efforts to rebalance and control the growth in health care spending [37, 41].
C. Establish and enforce primary care spending accountability mechanisms, including by:
  1. Developing and implementing a public dashboard of health plan and health system primary care spending, to foster transparency and encourage adherence. For example, the California Department of Health Care Access and Information (HCAI) recently announced plans to launch an interactive primary care dashboard, the Health of Primary Care in California Snapshot. The dashboard unites data from HCAI and other partners to provide comprehensive public reporting on primary care. HCAI has identified five areas of interest for assessment in the Snapshot: investment, workforce, access, quality, and equity. Each domain will include a concise set of primary care metrics to be reported annually [50].
  2. Empowering oversight and regulatory bodies to levy fines and develop health plan and health system performance improvement plans if primary care spending targets are not met.
  3. Requiring health systems to report on the flow of money earmarked for primary care (e.g., clinical revenue, capitation dollars, quality incentive payments) through the systems, to help ensure the funding reaches the intended targets (practices) rather than being diverted to other services.

Expert Committee members expressed concern that without such mechanisms in place, the impact of initiatives to increase primary care spending may be limited. For this reason, burgeoning U.S. state-level efforts to increase the proportion of health care spending on primary care are working to incorporate such approaches to attaining accountability at the health system and health plan levels [49, 51].

### Recommendation 2

Pay primary care clinicians and practices using models that support care teams in delivering high quality, equitable, relationship-centered primary care. This recommendation stemmed from the realization that the prevailing fee for service model of payment in the U.S. is insufficient to support robust primary care consistent with the NASEM definition. Fee for service payment is in essence reimbursement only for the services provided by primary care physicians and advanced practice professionals (e.g., nurse practitioners, physician assistants, and psychologists), not for the services of other important care team members such as social workers, dieticians, clinical pharmacists, and community health workers liaisons.

#### Sub-recommendations

A. Develop and implement hybrid payment models in which a majority of payment is provided as per member per month capitation and a lesser proportion as fee-for-service payment. Committee members cited experience suggesting that such hybrid models would allow for appropriate resourcing of robust primary care teams, supporting the critical work of all team members as well as supporting information and communications technology and other facilitating administrative support elements. Research also suggests such an approach is necessary to incentivize practices to adopt advanced primary care models [52].
  1. Ensure hybrid payment models are risk-adjusted to guide the determination of appropriately sized patient panel sizes for clinicians, and to ensure there is sufficient support for care teams designed to meet the needs of the populations served. The number of patients assigned (i.e., patient panel size) to primary care clinicians caring for more medically complex or “sicker” patient populations would ideally be smaller than for physicians with less complex patient populations, all other practice characteristics being held equal. Yet providing primary care for more complex patients tends to consume relatively more time and resources (e.g., office visits, ancillary services) per patient than caring for less complex individuals. Failure to include appropriate risk adjustment in hybrid payment models would unfairly penalize primary care clinicians and practices with smaller yet more complex patient panels.
  2. Consider unique hybrid payment models for three categories of practices: small, independent practices; community clinics (e.g., Federally Qualified Health Centers); and larger practices within integrated networks. This sub-recommendation stemmed from the recognition that supporting the adoption of expanded primary care services under hybrid payment, such as integrated behavioral health or social work services, would require different approaches across these practice types. For example, smaller practices may be unable to provide such services directly, but a hybrid model might be devised to incentivize their membership in networks of smaller practices to provide such services. By contrast, practices within large vertically integrated organizations may be well-positioned to adopt such expanded services quickly and could be incentivized through payment model incentives related to care quality or access metrics.
B. Ensure a reliable and meaningful means of attributing patients to primary care practices. Committee members agreed this recommendation is critical to hybrid payment models with per-member per-month capitation. However, it will require careful attention, since accurate patient attribution is difficult to achieve in practice, there is no consensus on the optimal methodology, and different methods yield widely different results [53].
C. Consider establishing state primary care stabilization funds, funded by all payers and sequestered from the larger health system budget, to fund all primary care services. Some Committee members felt that funding of primary care services should be accomplished via a completely different approach than for other types of health care, given its unique goals of delivering comprehensive, coordinated, and continuous care and the notion that it should be treated as a social good rather than a commodity [1, 4]. In Massachusetts, for example, advanced legislative efforts have been underway for several years to improve investment in primary care, including by establishing a state-administered primary care stabilization fund or “trust” [54]. The fund would be created from mandatory contributions from all health plans in the state and would administer monthly prospective payments (i.e., not retrospective fee for service payments) directly to primary care practices. More recently, the California Academy of Family Physicians’ Primary Care for All Task Force endorsed a similar approach and is engaging with various stakeholders in anticipation of future related legislation [55].
  Among other benefits, the state primary care stabilization fund approach would ensure alignment among all health plans in a region in efforts to increase the primary care investment, essential to realizing targeted improvements in population health and to slowing growth in total health care spending [56]. State primary care stabilization funds also would provide a dependable up-front funding stream for practices, facilitating investment in robust primary care models and teams, consistent with the NASEM blueprint. It would also greatly reduce the heavy administrative burden of dealing with multiple payers (each with their own unique rules and requirements), thereby reducing administrative overhead costs and allowing more resources to be utilized for patient care.
D. Develop a more objective and valid methodology for the valuation of medical services than the current system based on Relative Value Units (RVUs). As indicated by Sub-recommendation 2.A., there was strong consensus among Expert Committee members that current predominantly RVU-driven primary care payment models should be replaced by hybrid models in which most of the payment is via per-member, per-month capitation. However, the Committee members also acknowledged that at least over the short term, RVU based payment will continue to have a substantive role even in hybrid models, and that the current U.S. CMS methodology severely undervalues most primary care services [15]. Just as importantly, the current methodology also overvalues procedural (largely subspecialty) services, contributing to unsustainably high and rising total health care expenditures.

### Recommendation 3

Assist practices in transformation to advanced primary care models and assess the impacts on clinicians, practices, patients, and communities. Expert Committee members recognized the large amount of work and resources it can take to attain advanced primary care models, and that most practices currently fall well short of ideal primary care delivery.

#### Sub-recommendations

A. Incentivize and hold practices and health systems accountable for delivering high value primary care while minimizing the reporting burden by:
  1. Prioritizing monitoring of over-arching metrics that reflect core attributes of primary care including:
    ▪ Patient access to care via multiple modalities (e.g., in-person visits, asynchronous and synchronous telehealth, artificial intelligence [AI] platforms).
    ▪ Continuity, comprehensiveness, coordination, and patient-centeredness of care.
    Such incentives will help to ensure that practices pursue and maintain excellence in these core attributes of primary care, which are vital to reaping its full benefit on health outcomes including mortality [42, 43].
  2. Selecting and monitoring a manageably small number of condition- focused quality metrics that also reflect core primary care attributes. For example, related to depression:
    ▪ Services available (e.g., presence of on-site mental health treatment, community mental health workers),
    ▪ Services received (e.g., percent of patients with depression that received treatment), and
    ▪ Effect on outcomes (e.g., percent of patients with depression who attained remission based on symptom scores).
    Currently practices are held accountable for dozens of “quality metrics,” which differ across health plans and patient subgroups, and are often chosen because the data is easy to obtain rather than reflecting strength in core primary care attributes or high value care elements. This adds a heavy administrative burden and cost to practices (and ultimately health plans and patients) in the U.S. relative to other highly developed countries, with no benefit (and potentially detriment) to population health or to controlling spiraling total health care spending [57].
B. Monitor the impact of advanced primary care practice at the broader community level (e.g., acute care utilization, mortality, cost of care). Ecological study data indicates that a higher per capita concentration of primary care physicians in a region is associated with benefits in longevity [2]. Following this recommendation would afford a more complete picture of how robust primary care might both benefit population health and slow the growth in total healthcare spending.
C. Provide supplemental funding and technical consultation and assistance to help practices effectively and rapidly implement advanced primary care models including:
  1. Training and leveraging extended primary care team members such as community health workers to increase the reach of practices and support health and wellness in the communities they serve,
  2. Adopting AI and other technologies to reduce clinician administrative burden around charting, coding, and clinical decision-making, and
  3. Integrating behavioral health services, of paramount importance given the level of unmet in the U.S. and the impracticality of meeting the need via other channels.
    This recommendation recognizes the baseline wide variation among practices in capacity to pursue and attain advanced primary care models including team members and technologies, and that the work of transformation is both difficult and entails substantial up-front investment. State regulatory bodies, health systems, and perhaps even health plans and purchasers of health insurance might collaborate to organize and incentivize relatively more advanced practices and their care team members to serve as expert transformation consultants. In bringing a higher proportion of practices in a region up to the standards of the NASEM aspirational definition of primary care, all parties would benefit.
D. Monitor well-being, burnout, and retention of all primary care team members.
  There was strong agreement on the importance of this recommendation, given the complex relationship between practice transformation and burnout, which may vary at different points in the transformation process and among different care team members [58]. There is also considerable variation in adaptive reserve among practices at baseline, with higher levels of adaptive reserve being associated with less burnout, underscoring the advisability of assessing and, when indicated, seeking to bolster reserve prior to initiating and planning major transformation efforts [59].

### Recommendation 4

Maximize the impact of primary care as a lever for equitably advancing population health.

#### Sub-recommendations

A. Establish universal population health goals and employ multiple strategies in communicating and pursuing them, targeted to specific health system and societal structural barriers that adversely affect health and health care but to different degrees and ways across diverse groups of patients. Such an approach has advantages over the commonly employed conceptual frame for equity issues focused on closing gaps between groups that are faring better and those faring worse. Among other problems, such an approach can create tension and competition for attention and resources between groups and preclude the formation of broad-based coalitions working for change. By contrast, the approach recommended by the Expert Committee explicitly acknowledges and accounts for the fact that all people are situated within non-neutral structures (cultural, social, physical) that unevenly distribute benefits and burdens among groups and interact in ways that produce differential outcomes and can foster inequities [60]. It also acknowledges that for most health care measures, including primary care access and quality, *all* groups have considerable room for improvement, and that a range of strategies will be required to target an array of structural impediments.
B. Develop, implement, and enforce mandates for all ambulatory practices (primary care and subspecialty care) to participate in the care of patients with Medicaid insurance
  1. Explore the provision of additional payments (e.g., determined by risk adjustment) to practices that disproportionately care for the medically underserved.
    Following these sub-recommendations would mirror approaches to care of the underserved in many comparable industrialized countries, in which all practices and payers participate substantively in such care [61]. Currently, care of Medicaid patients is highly concentrated within a small proportion of independent practices [62].
C. Remove cost sharing (copays and deductibles) for primary care services. While the findings regarding the effects of patient cost-sharing on health care utilization and outcomes are mixed overall, there is clear evidence of net detrimental impacts on utilization and outcomes among low-resource individuals [63].
D. Ensure that risk adjustment methodologies account for both clinical factors and social influences on health. Accounting for social influences in risk adjustment is important. Although effective health care system interventions to account for and address social influences are still in evolution, such factors account for far more of the variance in health care utilization and outcomes among individuals than do clinical indicators [64].
E. Ensure adequate information technology and infrastructure (e.g., data exchange capabilities between relevant entities) to support ongoing measurement, monitoring, and public reporting of health equity-related data (e.g., primary care access, health disparities). Attaining this recommendation will be challenging given the patchwork nature of our health care “system,” yet will be vitally important to ensure that primary care revitalization is having its intended positive impacts in all groups, consistent with a Targeted Universalism frame [60].

### Recommendation 5

Advocate for the training of an appropriately-sized and diverse primary care physician workforce.

#### Sub-recommendations

A. Advocate for:
  1. Centers for Medicare and Medicaid Services to develop and publicly report on adherence to policies that ensure distribution of graduate medical education (GME) funding is proportionate to regional community needs,
  2. State governmental use of Medicaid funds to support GME in ways that better meet the needs of all communities, such as training in ambulatory facilities (e.g., Federally Qualified Health Centers) in rural and urban medically underserved areas, and
  3. Community governance structures that hold Medicare and other GME funders (e.g., states) accountable for ensuring that primary care workforce needs are met in all regions.

No such GME accountability measures have ever been put in place in the U.S. This fundamental failure of planning and oversight remains a major contributor to the mismatches in the distribution of specialties among newly minted physicians and the places they choose to practice versus the needs of the population [26, 27].

### Recommendation 6

Expand resources and infrastructure to facilitate research addressing questions of pressing relevance to primary care and its revitalization. The Expert Committee asserted that the existing primary care research base and data sets have established clear benefits of greater patient and population level exposure to primary care – including but not limited to longer life expectancy/reduced mortality, less disability, and more receipt of evidence-based preventive care services [2, 42, 43]. Research also has shown the beneficial effects of core primary care values, processes, and structures on equity outcomes [3].

Specific to data sets, the Expert Committee also noted the growth of the PRIME registry and other American Board of Family Medicine databases [65], alongside other electronic health record data and all payer claims databases that can be leveraged for more primary care research opportunities. and several prior and ongoing research funding mechanisms offered by federal and private foundation sources [66].

Nonetheless, many highly pressing questions in primary care remain unanswered, in part due to the often-complex nature of the questions and the lack of corresponding resources and infrastructure to enable rigorous studies to address them. The Expert Committee noted that a major contributor to this problem is the fundamental mismatch between the nature of the most pressing primary care research gaps and the stated priorities and funding opportunities of the National Institutes of Health and other major funders of health care research in the U.S. While there are a few prior and ongoing research funding mechanisms specific to primary care offered by federal and private foundation sources [66], these remain an exception, with less than 1% of all federal research dollars devoted to investigating primary care [7]. New interdisciplinary funding priorities will be needed to support research capable of capturing the broad impact of primary care.

#### Sub-recommendations

A. Increase federal and other funding to support primary care research, with a focus on new funding streams to support rigorous examination of topics that reflect the full complexity and cross-cutting nature of primary care.
B. Broaden the reach, scope, applicability, and impact of primary care research by developing:
  1. Hub and spoke geographic networks of participating practices and investigators co-designing research studies, and
  2. A national longitudinal registry of primary care practices and core practice data elements to support larger scale collaborations and research.
    Sub-recommendations B.1 and B.2 are particularly important given the wide variation in characteristics among practices in the U.S. and because studies involving only one or a few practices within a single geographic region have limited power and generalizability. Attaining these sub-recommendations would enable a broader understanding of the current state of primary care practice, informing more broadly salient research, so that the most prevalent and important issues are addressed with more robust and broadly generalizable findings.
C. Develop and disseminate robust gold standard measures reflective of primary care attributes and elements – such as patient-centeredness, primary care team-centeredness, and primary care practice characteristics (e.g. patient panel sizes), to increase validity within studies and comparability across studies.
D. Develop and consistently apply more advanced analytic approaches (e.g., parallel mixed methods, rigorous risk adjustment) better suited to the complexity of primary care and related research questions.
E. Build more effective strategies to disseminate primary care research evidence into clinical practice. An example of an effective strategy is the Primary Care Extension Program authorized by the Affordable Care Act and tested by the Agency for Healthcare Research and Quality [67].

### Recommendation 7

Engage, educate, and collaborate with a broad array of societal stakeholders in messaging the vital importance of robust primary care to population health and health equity. There was broad agreement among Expert Committee members on the need for greater clarity, strength, and frequency of communication with a comprehensive array of societal stakeholders regarding primary care: what it looks like in its ideal state; how it can benefit individual and population health.; and how chronic under-resourcing is greatly limiting its impact, to the great detriment of public health. Prior research suggests a fundamental lack of understanding of at least some of these issues among the public [68]. Several of the sub-recommendations following from this recommendation entail promising innovative approaches.

#### Sub-recommendations

A. Engage, educate, and facilitate the building of coalitions among the various stakeholders in a strong U.S. primary care base.
B. Develop a robust communication strategy which clearly conveys that *all* in the U.S. are poorly served by primary care in its current state, though some groups and communities are more poorly served than others.
C. Create and disseminate a library of evidence-based primary care marketing resources via a hub and spoke model, to provide more consistent and powerful messaging about the importance of primary care to the U.S. population.
D. Consider efforts to increase health and primary care literacy at the population level (e.g., via inclusion of elements in standard elementary through high school curricula).
E. Strongly encourage community governance structures for all primary care practices.

## Discussion

The recommendations in this report resulted from the activities of the Summit to Revitalize Primary Care (Rev PC), which facilitated deliberations among approximately 30 national experts and thought leaders in primary care. Most of our recommendations have some overlap with the five “implementation objectives” of 2021 NASEM ad hoc committee on primary care and subsequently appointed Standing Committee on Primary Care [1, 69]. Such overlap is warranted, given that five years after the NASEM report its implementation objectives remain aspirational, none having been widely achieved in the U.S. The Rev PC recommendations expand on the NASEM objectives by incorporating concrete suggestions for holding health plans and health systems accountable to increased primary care spending targets and graduate medical education funders to training an appropriate physician workforce; optimizing primary care funding models and helping practices in transformation to advanced primary care; ensuring equitable primary care access; and expanding primary care research infrastructure and funding. Another Rev PC addition is to signal the importance of and outline specific strategies for educating the public on the critical role of primary care to health (Recommendation 7), a topic not addressed by the NASEM objectives.

Several limitations of the Rev PC Summit efforts must be acknowledged. Our Expert Committee brought to bear hundreds of years of collective and relevant experience. Still, their recommendations largely reflect thinking on current best practices stemming from combined wisdom and limited or preliminary scholarly work, rather than firmly evidence-based strategies. As reflected in Recommendation 6 in this report, there remains a pressing need for research and evaluation projects to examine the abilities of governmental entities, regulatory bodies, health systems and practices to successfully pursue the recommendations. As recommendations are successfully pursued, there will be need to study the effects of implementation on care team and patient outcomes, population health, and health care spending. It is hoped this report will spur such projects and prompt funders to develop mechanisms to support them.

The Nominal Group Technique employed in two of the four Expert Committee working sessions is a well-described and widely used approach to generating consensus. The approach was well suited to our needs, given that the committee members convened in person and were available for only a limited time. With more committee member time available and full anonymity, the Delphi method might have been employed, though it has its own limitations [40]. The other two Rev PC Expert Committee working sessions employed somewhat less structured facilitation, though with attention to soliciting input from all members. We did not direct the Expert Committee members to identify targeted stakeholder or “actor” groups for the recommendations they generated. Nonetheless, they did identify targeted entities in some of the recommendations, and we believe most have implications for numerous stakeholder groups. Lastly, by design given the focus and aims of the Summit, the Expert Committee did not have representation from some stakeholders in revitalized primary care: patients, consumer advocates, legislators, and philanthropists. Prior complementary revitalization efforts have fruitfully engaged some of these groups and their voices will continue to be important in future work [3].

## Conclusion

We hope the recommendations in this report will be disseminated widely to further accelerate the growing momentum toward appropriately supporting primary care —the foundation of health care. Only in this manner will primary care finally achieve its vast yet unrealized potential to equitably advance population health while helping to control escalating health care costs. The human and financial health of the U.S. depends on healthy primary care.

## Data Availability

All the plenary presentations from the Summit and summaries of the conduct of the closed Expert Committee working sessions are available on the Summit website, with the participants’ approval—https://health.ucdavis.edu/family-medicine/news-events/optimizing-the-primary-care-spend-symposium/.

## Abbreviations

AI: Artificial intelligence
CMS: U.S. Centers for Medicare and Medicaid Services
GME: Graduate medical education
HCAI: California Department of Health Care Access and Information (HCAI)
NASEM: National Academies of Science, Engineering, and Medicine
OHCA: California Office of Healthcare Affordability
Rev PC: The Summit to Revitalize Primary Care
RVU: Relative Value Unit
UCD: University of California Davis
U.S.: United States
